# Adopted walking condition for computational simulation approach on bearing of hip joint prosthesis: review over the past 30 years

**DOI:** 10.1016/j.heliyon.2022.e12050

**Published:** 2022-12-05

**Authors:** J. Jamari, Muhammad Imam Ammarullah, Gatot Santoso, S. Sugiharto, Toto Supriyono, Muki Satya Permana, Tri Indah Winarni, Emile van der Heide

**Affiliations:** a Department of Mechanical Engineering, Faculty of Engineering, Diponegoro University, Semarang 50275, Central Java, Indonesia; b Undip Biomechanics Engineering & Research Centre (UBM-ERC), Diponegoro University, Semarang 50275, Central Java, Indonesia; c Department of Mechanical Engineering, Faculty of Engineering, Pasundan University, Bandung 40153, West Java, Indonesia; d Biomechanics and Biomedics Engineering Research Centre, Pasundan University, Bandung 40153, West Java, Indonesia; e Department of Anatomy, Faculty of Medicine, Diponegoro University, Semarang 50275, Central Java, Indonesia; f Center for Biomedical Research (CEBIOR), Faculty of Medicine, Diponegoro University, Semarang 50275, Central Java, Indonesia; g Department of Mechanics of Solids, Surfaces & Systems (MS3), Faculty of Engineering Technology, University of Twente, P.O. Box 217, 7500 AE Enschede, the Netherlands; h Laboratory for Surface Technology and Tribology, Faculty of Engineering Technology, University of Twente, Postbox 217, 7500 AE Enschede, the Netherlands

**Keywords:** Computational simulation, Human hip joint, Hip joint prosthesis, Hip resurfacing, Total hip arthroplasty, Walking condition

## Abstract

Bearing on artificial hip joint experiences friction, wear, and surface damage that impact on overall performance and leading to failure at a particular time due to continuous contact that endangers the user. Assessing bearing hip joint using clinical study, experimental testing, and mathematical formula approach is challenging because there are some obstacles from each approach. Computational simulation is an effective alternative approach that is affordable, relatively fast, and more accessible than other approaches in examining various complex conditions requiring extensive resources and several different parameters. In particular, different gait cycles affect the sliding distance and distribution of gait loading acting on the joints. Appropriate selection and addition of gait cycles in computation modelling are crucial for accurate and reliable prediction and analysis of bearing performance such as wear a failure of implants. However, a wide spread of gait cycles and loading data are being considered and studied by researchers as reported in literature. The current article describes a comprehensive literature review adopted walking condition that has been carried out to study bearing using computational simulation approach over the past 30 years. Many knowledge gaps related to adoption procedures, simplification, and future research have been identified to obtain bearing analysis results with more realistic computational simulation approach according to physiological human hip joints.

## 1 Introduction

**Figure 1:**
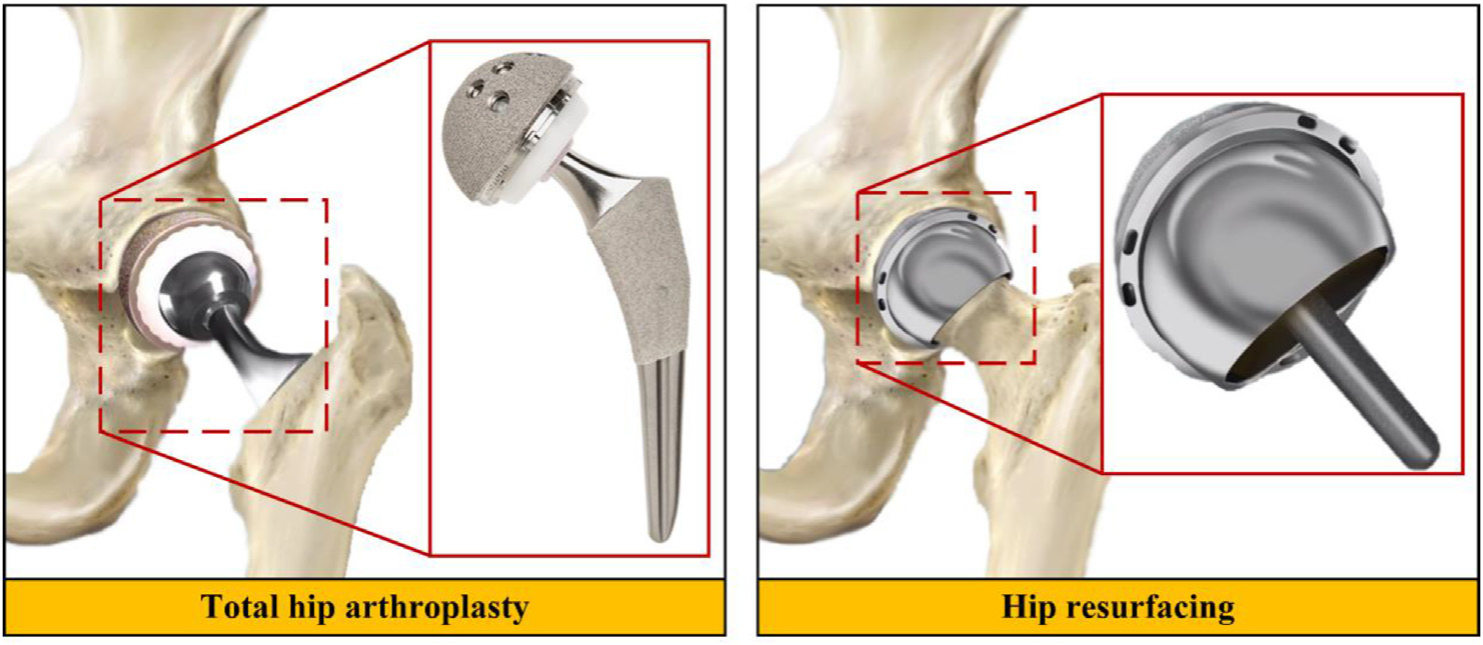
Total hip arthroplasty and hip resurfacing.

Hip joint replacement is a medical procedure conducting physical replacement of damaged human hip joint with hip joint prosthesis. This surgery has been considered the best option to relieve damage human hip joint to reduce pain, enhance joint functional, and improve life quality of patients [1, 2, 3]. This operation has been performed more than one million times worldwide since 2005 and is expected will be increased until 2030, because many people are getting older who experience various health problems, especially in their hip joints and require medical treatment to carry out normal daily activities as before [4]. There are two procedures in hip joint replacement surgery described in Figure 1, namely total hip arthroplasty and hip resurfacing [5, 6, 7]. The first procedure involves replacing femur head, femoral stem, and acetabulum cup, while the second procedure does not involve replacing femoral stem with implant component.

Although hip joint replacement surgery is considered one of the biggest developments in orthopaedics in the last few decades, this operation has not been entirely successfully studied from a mechanical perspective, so it requires further studies. The service life of hip joint prosthesis is generally limited to range of 15 years that does not provide satisfaction for young patients under 60 years of age with longer life expectancy, more than 40% expect implant life to exceed 20 or 25 years [8]. The success of replacement operation depends largely on hip joint implant quality. Therefore, various studies related to artificial hip joints have attempted to improve capabilities, both medical [9] and mechanical [10].

In implant's components, the bearing couple that consist of femoral head and acetabular cup play an essential role as load-bearing and provide movement articulation that continuously in contact for every user's activity [11]. Bearing couples can experience friction, wear, and surface damage affecting overall performance and lead to failure at certain time [12]. For avoid failure of medical implants that could be harmful for patient, various studies in bearing couple have attempted to ensure that implant bearings can last a long time to minimize implant failure or no revision surgery is required in the future [13, 14, 15].

In performing various bearing couple studies on medical implant, four approaches can be used, namely clinical study [16], experimental testing [17], mathematical formula [18], and computational simulation [19]. In the first approach, the method used are radiographic [20], computed tomography [21], and hip analysis suite [22]. Bearing couple studies involving implant users are the most realistic approach of providing valuable results according to actual daily human activities because they are carried out directly under physiological conditions. Unfortunately, without active participation from patients during conducted study, research with this approach could not provide meaningful results. The second approach is achieved by experimental tools, such as hip joint simulator [23], pin-on-disc [24], pin-on-plane [25], ball-on-disc [26], and ball-on-plane [27]. Experimental testing requires sophisticated and high-cost equipment becomes major obstacle for many researchers. The third approach uses analytical mathematical formula based on contact mechanics [28], fluid mechanics [29], and biotribology [30]. Mathematical formulation is the basic concept for many researchers conducting various studies on bearing of hip joint prostheses, but solving realistic problems is very difficult using this approach and is prone to miscalculations because it is done manually or solved numerically.

Computational simulation approach enables to overcome the most common problems found in the first three approaches. This approach carried out by various researchers currently uses finite element method to investigate bearing couple on hip joint arthroplasty with various parameters for further exploration [31, 32, 33]. It is very possible to do, where this approach provides efficiency in time, difficulty, and cost compared to previous three approaches. Analyse using finite element method can also be investigated as preliminary research in assessing various problems. After obtaining results from computational simulation, study can be continued to experimental testing or clinical study approaches [34, 35, 36, 37]. With the current development of software technology, mathematical formula approach has been further developed in finite element-based computational simulation, thus making the current mathematical formula less desirable.

Over the past 30 years, various efforts to develop bearing couples of artificial hip joints by many researchers using computational simulation presented in this review paper have been studied various aspects, such as geometry [38], materials [39], lubrication [40], textured surface [41], and coatings [42]. In computational study of hip joint implant's bearing, two domains can be studied, the first is solid domain representing femoral head and acetabular cup component and the second is fluid domain representing synovial body fluid. The research was conducted by looking for results on solid domain, there are contact pressure [43], wear [44], von Mises stress [45], sliding track [46], heat [47], cross-shear [46], displacement [48], plastic strain [49], Tresca stress [50], creep [51], principal stress [52], and equivalent strain [53]. Furthermore, on fluid domain, there are fluid pressure [41], hydrodynamic pressure [54], film thickness [55], and eccentricities [56]. The results have been obtained through computational simulation approach and then further analysed with various theories, followed by comparisons with rational explanation.

The objective of present review article is to comprehensively summarizes the adoption of walking conditions in previous studies using computational simulations to assess couple bearing in total hip prosthesis and hip resurfacing. The previous literature over the past 30 years (1992–2022) from Scopus database has been collected and further examined to understand adopted walking conditions that have been done using the computational simulation approach. In-depth information about adoption procedures, simplification, and future research has been presented in this review for filling knowledge gaps in the literature.

## 2 Adopted walking condition for computational simulation approach

Computational simulation based on finite element on bearing of hip joint prosthesis have been attempted to represent how bearing working in actual condition, when used by patient, so this approach can not give significant difference on results from clinical study or experimental testing. Therefore, it is necessary to establish a variety of input data and boundary conditions that are as realistic as possible to achieve results that are closer to actual condition. The use of realistic loading originating from physiological of human hip joint has been carried out in various previous studies by adopting physiological this joint in performing various activities. With rationalization walking is the most common human activity, most realistic loading considered by many researchers to study bearing of hip joint implant is adoption of walking condition [47, 53, 57, 58, 59].

**Figure 2:**
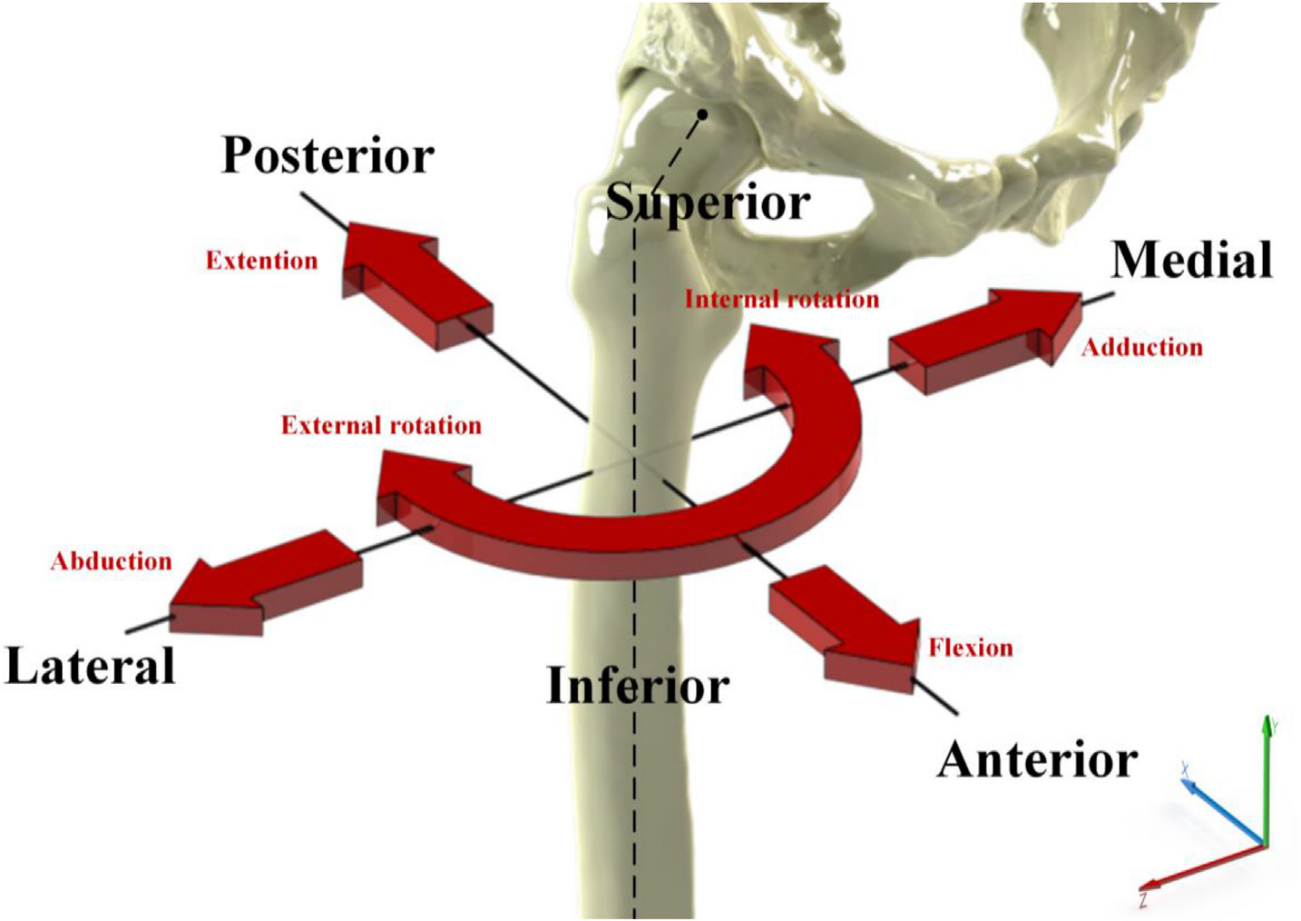
Loading and motions in human hip joint.

**Figure 3:**
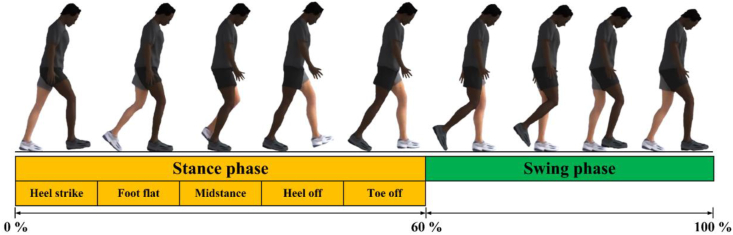
Phase description in walking cycle. Maximum loading acted on foot flat (stance phase) and minimum loading acted on throughout swing phase.

With regards to physiological human hip joint during walking, there are loading and motions that work in three dimensions, shown in Figure 2. Loading acts on x-, y-, and z- axes, which form a resultant force. Also, there are motions from femur relative to pelvis at three degrees of freedom, namely flexion-extension (FE), abduction-adduction (AA), and internal-external rotation (IER). It can be seen that FE moves on x-axis (sagittal plane), IER moves on y-axis (transverse plane), and AA moves on z-axis (coronal plane) [60]. When performing walking condition, movement along three axes is based on time period from heel strike to next heel strike of the same leg, known as cycle [61]. Walking cycle, presented in Figure 3 is divided into two major groups: stance phase (phase where feet come into contact with the ground during walking) and swinging phase (phase where feet swing freely during walking). The stance phase is classified into five sub-phases: heel strike, foot flat, midstance, heel-off, and toe-off [62]. Resultant force that can work received by hip joint depends on body weight and muscle strength estimated to be around three times body weight during walking condition [63]. In this condition, the maximum resultant force is on foot flat and minimal during swing phase [64].

In adopting walking condition, three components must be considered for computational simulation approach, there are loading, motions, and cycle described in Table 1. Adoption type of loading consists of 3D load, 2D load, 1D load, and static load. Also, motions component consists of FE-AA-IER, FE-AA, FE-IER, and FE. Then, cycle component consists of full cycle, mid-to-terminal stance, stance phase, peak loading, specific conditions, and divided.

**Table 1:** Adopted walking condition for computational simulation approach on bearing of hip joint prosthesis.

Components	Adoption type	Description
Loading	3D load	Adopted physiological human hip joint loading from x-, y-, and z-axes
	2D load	Only adopted human hip joint loading from x- and y-axes
	1D load	Only adopted human hip joint loading from y-axis
	Static load	3D/2D/1D load, but only adopted peak loading/specific condition on cycle
	No-load	Not adopted human hip joint loading
Motions	FE-AA-IER	Adopted physiological human hip joint motion on FE, AA, and IER
	FE-AA	Only adopted human hip joint motion on FE and AA
	FE-IER	Only adopted human hip joint motion on FE and IER
	FE	Only adopted human hip joint motion on FE
	No motion	Not adopted human hip joint motion
Cycle	Full cycle	Adopted full walking cycle
	Mid-to-terminal stance	Only adopted mid-to-terminal stance under walking cycle
	Stance phase	Only adopted stance phase under walking cycle
	Peak loading	Only adopted peak loading under walking cycle, also without motion
	Specific condition	Only adopted specific phase under walking cycle, also without motion
	Divided	Adopted walking cycle (full or partial) divided into many phases

**Figure 4:**
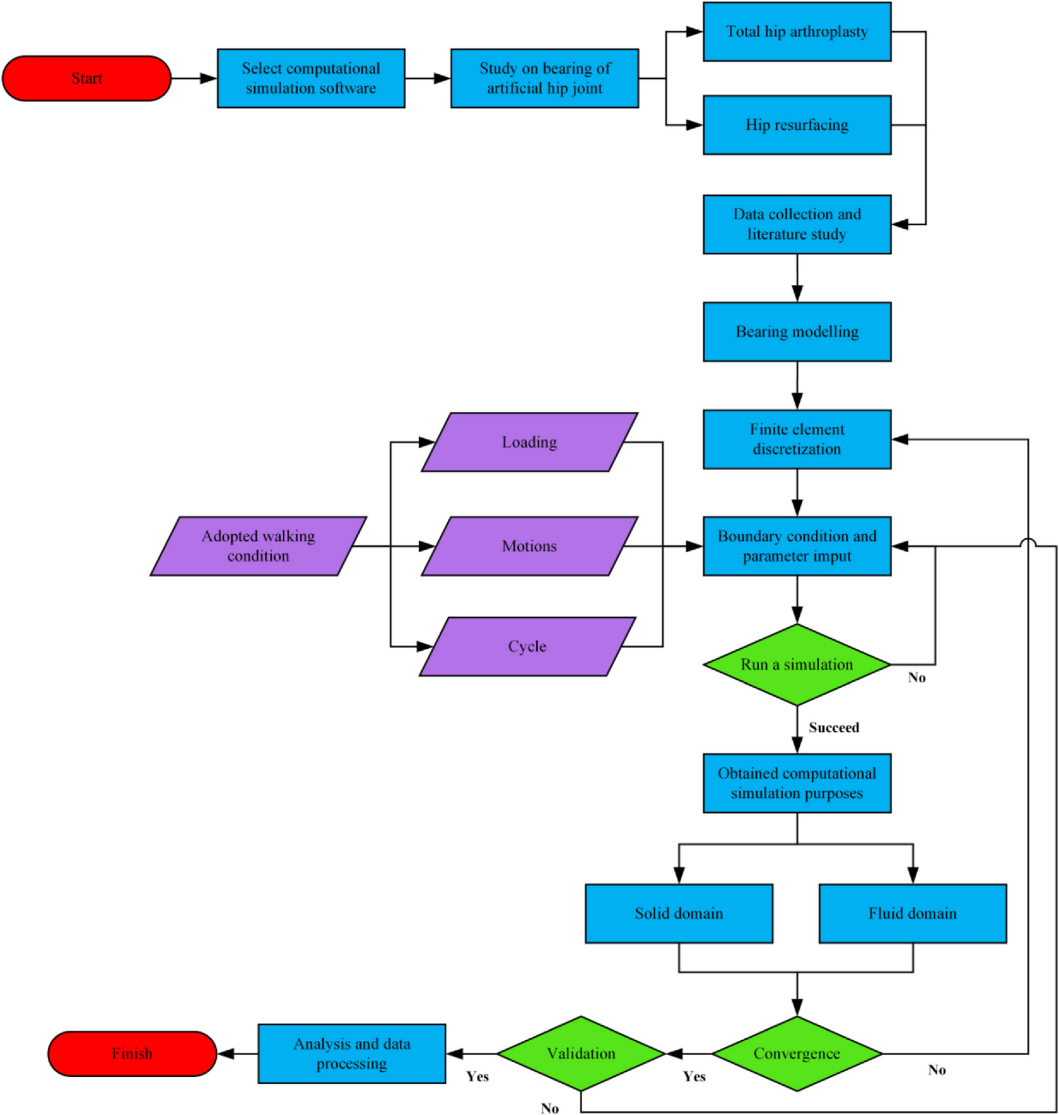
Overview of computational simulation study on bearing of hip joint prosthesis that adopted walking condition.

In general, research flow of computational simulation approach using finite element method on artificial hip joint's bearing under walking conditions is described in Figure 4. First, bearing model of the hip joint implant is made, then finite element discretization is analysed. When input parameters and boundary conditions, adoption of walking conditions is operated according to computational simulation purposes, either according to physiological human hip joint or considering simplification on walking cycle components. Second, computational simulation run to obtain purposes at solid, fluid, or both domains. Lastly, obtained data were studied for further analysis.

## 3 Computational simulation study on bearing of hip joint prosthesis under walking condition

In computational simulation approach to assess bearing on hip joint prosthesis, two aspects affect the results under walking conditions, there are adopted walking condition and walking condition reference. In first aspect, it is divided into simplified and physiological walking conditions. For the second aspect, it is divided into ISO, published literature, and independent measurement. Therefore, it will not be able to get the same simulation results from one researcher to another, even examining same type of hip joint bearing under walking conditions in computational simulation studies. For more detailed, it is described in Table 2.

**Table 2:** Aspects that affect computational simulation results on bearing of hip joint implant under walking condition.

Aspect	Adoption type	Description
Adopted walking condition	Simplified walking condition	Using walking condition with simplification from any components in loading, motions, and cycle that not corresponds to physiological human hip joint
	Physiological walking condition	Adopted walking condition without any simplification from any components in loading, motions, and cycle that corresponds to physiological human hip joint
Walking condition reference	ISO	Using walking condition presented by International Organization for Standardization
	Published literature	Using walking condition presented by others researchers from published literature
	Independent measurement	Using walking condition obtained independently along with conducted computational simulation study

Over the past 30 years, many researchers have conducted several efforts to develop bearing of hip joint arthroplasty using the finite element method under walking condition. Several computational simulation software selected to conduct finite element analysis, like ANSYS [57], ABAQUS [65], COMSOL Multiphysics [38, 54], MATLAB [44], Adams [66], and AnyBody Modelling System [67]. Both bearings of total hip arthroplasty and hip resurfacing have been studied, but mostly doing investigations on total hip arthroplasty [68, 69, 70]. Computational studies have been investigated to obtain results, whether on solid, fluid, or both domains. The adopted walking condition has also been observed further along with its components, including loading, motions, and cycle. The detail of computational simulation previous studies on a bearing of hip joint arthroplasty under walking condition adopted from Scopus database over the past 30 years (1992–2022) has been summarized in Table 3.

**Table 3:** Summary of detail computational simulation studies on bearing of hip joint prosthesis that adopted walking conditions over the past 30 years.

Authors	Computational simulation software	Type of hip joint prosthesis	Computational simulation purposes	Adopted walking condition	Walking condition components					Variation of adopted walking/others condition
					Loading		Motion		Cycle	
					Type	Reference	Type	Reference		
Affatato et al. [57]	ANSYS v. 18.1	Total hip arthroplasty	Contact pressure	Physiological walking condition	3D load	Damsgaard et al. [71]	FE-AA-IER	Damsgaard et al. [71]	Full cycle	−
Ammarullah et al. [59]	ABAQUS	Total hip arthroplasty	Contact pressure and wear	Simplified walking condition	3D load	Bergmann et al. [63]	No motion	−	Full cycle divided into 32 phases	−
Ammarullah et al. [50, 72]	ABAQUS v. 6.14–1	Total hip arthroplasty	Tresca stress	Simplified walking condition	3D load	Bergmann et al. [63]	No motion	−	Full cycle divided into 32 phases	
Barreto et al. [73]	ABAQUS v. 6.7	Total hip arthroplasty	Contact pressure and wear	Simplified walking condition	3D load	Bergmann et al. [63]	No motion	−	Full cycle divided into 28 phases	−
Basri et al. [38, 54]	COMSOL Multiphysics v. 4.3b	Total hip arthroplasty	contact pressure, hydrodynamic pressure, and film thickness	Simplified walking condition	1D load	ISO 14242–1:2012 [74]	No motion	−	Full cycle	−
Brown et al. [75]	ABAQUS	Total hip arthroplasty	Contact pressure and wear	Simplified walking condition	3D load	Brand et al. [76]	FE	Brand et al. [76]	Full cycle	−
Cilingir [77]	ABAQUS v. 6.9	Hip resurfacing	Von Mises stress and contact pressure	Simplified walking condition	Static load	Bergmann et al. [63]	No motion	−	Peak loading	−
Cilingir et al. [78]	ABAQUS v. 6.5	Hip resurfacing	Contact pressure	Simplified walking condition	Static load	Bergmann et al. [79]	No motion	−	Peak loading	−
Fialho et al. [47]	ANSYS	Total hip arthroplasty	Contact pressure, wear, and heat generation	Simplified walking condition	3D load	Bergmann et al. [63]	FE-AA-IER	Bergmann et al. [63]	Full cycle divided into 28 phases	−
Gao et al. [41]	Not mentioned	Total hip arthroplasty	Fluid pressure and film thickness	Simplified walking condition	1D load	ISO 14242–1:2002 [74]	FE	ISO 14242–1:2002 [74]	Full cycle	−
Gao et al. [43]	ABAQUS v. 6.12	Total hip arthroplasty	Contact pressure	Simplified walking condition	3D load	Paul [64]	FE-AA-IER	Johnston and Smidt [80]	Full cycle divided into 41 phases	−
Gao et al. [56]	Not mentioned	Total hip arthroplasty	Fluid pressure, film thickness, and eccentricities	Simplified and Physiological walking condition	1D load and 3D load	Bergmann et al. [63]	FE and FE-AA-IER	Bergmann et al. [63]	Full cycle	Walking, stairs up/down, and stand up/sit down
Harun et al. [68]	ABAQUS v. 6.53	Total hip arthroplasty	Contact pressure and wear	Simplified walking condition	1D load	Paul [64]	FE-AA-IER	Johnston and Smidt [80]	Full cycle divided into 20 phases	−
Heijink et al. [45]	ABAQUS	Hip resurfacing	Von Mises stress	Simplified walking condition	Static load	Bergmann et al. [63]	No motion	−	Peak loading	−
Hua et al. [81]	ABAQUS v. 6.9	Total hip arthroplasty	Contact pressure and von Mises stress	Simplified walking condition	Static load	Udofia et al. [82]	No motion	−	Peak loading	−
Jamari et al. [83]	ABAQUS v. 6.14–1	Total hip arthroplasty	Contact pressure and wear	Simplified walking condition	3D load	Bergmann et al. [63]	FE-AA-IER	Bergmann et al. [63]	Full cycle divided into 32 phases	−
Jamari et al. [65, 84]	ABAQUS v. 6.14–1	Total hip arthroplasty	Contact pressure	Simplified walking condition	3D load	Bergmann et al. [63]	No motion	−	Full cycle divided into 32 phases	−
Jourdan and Samida [44]	MATLAB	Total hip arthroplasty	Wear	Physiological walking condition	3D load	Paul [64]	FE-AA-IER	Johnston and Smidt [80]	Full cycle	-
Kang et al. [46]	MATLAB v. 7.0	Total hip arthroplasty	Contact pressure, sliding track. Cross-shear, and wear	Physiological walking condition	3D load	Paul [64]	FE-AA-IER	Johnston and Smidt [80]	Full cycle	
Krepelka and Toth-Taşcău [70]	ANSYS	Total hip arthroplasty	Contact pressure	Simplified walking condition	Static load	Bergmann et al. [63]	No motion	−	Peak loading	Walking and stairs up/down
Liu et al. [85]	ABAQUS v. 6.8–1	Total hip arthroplasty	Wear, contact pressure, and cross-shear	Physiological and simplified walking condition	1D and 3D load	Johnston and Smidt [80] (3D load), ISO 14242–1:2002 [74] (1D load), and ProSim hip joint simulator [86] (1D load)	FE-AA-IER, FE-AA-IER, and FE-IER	Johnston and Smidt [80], ISO 14242–1:2002 [74], and ProSim hip joint simulator [86]	Full cycle	Three different walking condition from Johnston and Smidt [80], ISO 14242–1:2002 [74], and ProSim hip joint simulator [86]
Liu et al. [87]	ABAQUS	Hip resurfacing	Contact pressure and wear	Simplified walking condition	1D load	Leslie et al. [88]	FE-IER	Leslie et al. [88]	Full cycle divided into 32 phases	−
Liu et al. [89]	Not mentioned	Hip resurfacing	Film thickness and film pressure	Simplified walking condition	1D load	ISO 14242–1:2002 [74]	FE	ISO 14242–1:2002 [74]	Full cycle	−
Liu et al. [90]	Not mentioned	Total hip arthroplasty	Film thickness and hydrodynamic pressure	Simplified walking condition	1D load	Rieker et al. [91]	FE	Rieker et al. [91]	Full cycle	−
Liu et al. [49]	ABAQUS v. 6.11–1	Total hip arthroplasty	Contact pressure and plastic strain	Simplified walking condition	Static load	ISO 14242–1:2002 [74]	No motion	−	Specific condition	Static load under walking condition with magnitude of 0.5 kN and 3 kN
Liu et al. [66]	Adams 2013	Total hip arthroplasty	Contact pressure	Simplified walking condition	1D load	ISO 14242–1:2002 [74]	FE-AA	ISO 14242–1:2002 [74]	Full cycle	−
Liu et al. [92]	ABAQUS v. 6.8–1	Total hip arthroplasty	Contact pressure, creep and wear	Simplified walking condition	1D load	ProSim hip joint simulator [86]	FE-IER	ProSim hip joint simulator [86]	Full cycle	−
Matsoukas and Kim [51]	MATLAB	Total hip arthroplasty	Contact pressure, creep, and wear	Physiological walking condition	3D load	Bergmann et al. [63]	FE-AA-IER	Bergmann et al. [63]	Full cycle	Walking and stairs up
Maxian et al. [69]	ABAQUS v. 5.3	Total hip arthroplasty	Contact pressure and wear	Simplified walking condition	3D load	Brand et al. [76]	FE	Brand et al. [76]	Stance phase divided into 16 phases	−
Maxian et al. [93]	ABAQUS v. 5.3	Total hip arthroplasty	Wear	Simplified walking condition	3D load	Mejia and Brierly [94]	FE-AA	Mejia and Brierly [94]	Full cycle	−
Mellon et al. [95]	MATLAB	Hip resurfacing	Contact pressure	Physiological walking condition	3D load	Bergmann et al. [63]	FE-AA-IER	independent measurement	Full cycle	Four different adopted motions
Meng et al. [48]	ABAQUS v. 6.7–1	Total hip arthroplasty	Contact pressure, displacement, fluid pressure, and film thickness	Simplified walking condition	1D load	−	FE	Bergmann et al. [63]	Peak loading	−
Meng et al. [96]	ABAQUS v. 6.9–1	Total hip arthroplasty	Contact pressure, film pressure, and film thickness	Simplified walking condition	1D load	ISO 14242–1:2002 [74]	FE	ISO 14242–1:2002 [74]	Full cycle	−
Nithyaprakash et al. [97]	ANSYS	Total hip arthroplasty	Contact pressure, principal stress and wear	Simplified walking condition	3D load	Bergmann et al. [63]	FE-AA-IER	Bergmann et al. [63]	Full cycle divided into 32 phases	Normal walking with peak load 2.41 kN, normal walking with peak load 3.327 kN, sitting down/getting up, carrying load 25 kg, carrying load 40 kg, stairs up/down, and ladder up/down (70°)
Onisoru et al. [98]	ANSYS	Total hip arthroplasty	Wear	Simplified walking condition	3D load	Bergmann et al. [63]	No motion	−	Full cycle	Normal walking and stairs up/down
Pakhaliuk and Poliakov [99]	ANSYS and MATLAB	Total hip arthroplasty	Wear	Simplified walking condition	2D load	ISO 14242–1:2012 [74]	FE-AA-IER	ISO 14242–1:2012 [74]	Full cycle divided into 25 phases	Walking, stairs up/down, standing up/sitting down, and deep squatting
Pakhaliuk [100]	ANSYS and MATLAB	Total hip arthroplasty	Wear	Simplified walking condition	2D load	ISO 14242–1:2012 [74]	FE-AA-IER	ISO 14242–1:2012 [74]	Full cycle divided into 25 phases	−
Patil et al. [101]	Marc	Total hip arthroplasty	Wear	Simplified walking condition	3D load	Brand et al. [76]	No motion	−	Full cycle	−
Peng et al. [58]	ABAQUS v. 6.12	Total hip arthroplasty	Wear	Physiological walking condition	3D load	independent measurement	FE-AA-IER	independent measurement	Full cycle	−
Raimondi [102]	ABAQUS	Total hip arthroplasty	Contact pressure and wear	Physiological walking condition	3D load	Bergmann et al. [63]	FE-AA-IER	Sutherland et al. [103]	Full cycle	−
Ruggiero et al. [67]	AnyBody Modelling System	Total hip arthroplasty	Contact pressure and wear	Physiological walking condition	3D load	Damsgaard et al. [71]	FE-AA-IER	Damsgaard et al. [71]	Full cycle	−
Saputra et al. [104]	ABAQUS	Total hip arthroplasty	Contact pressure and von Mises stress	Simplified walking condition	Static load	Paul [64]	No motion	−	Peak loading	-
Shankar et al. [105]	ANSYS v. 14.0	Total hip arthroplasty	Contact pressure and wear	Simplified walking condition	Static load	Bergmann et al. [63]	No motion	−	Full cycle divided into 32 phases	−
Shankar et al., [106]	ANSYS v. 14.0	Total hip arthroplasty	Contact pressure, von Mises stress, and wear	Simplified walking condition	3D load	Bergmann et al. [63]	FE-AA-IER	Bergmann et al. [63]	Full cycle divided into 32 phases	−
Shankar et al. [107]	ANSYS	Total hip arthroplasty	Contact pressure and wear	Simplified walking condition	3D load	Bergmann et al. [63]	No motion	−	Peak loading	−
Shankar et al. [108]	ANSYS	Total hip arthroplasty	Contact pressure and wear	Simplified walking condition	3D load	Bergmann et al. [63]	FE-AA-IER	Bergmann et al. [63]	Full cycle divided into 32 phases	Walking, lifting 40 kg, carrying 25 kg, stairs down 25 kg, ladder up 70^o^/90^o^, and ladder down 70^o^/90^o^
Suri et al. [109]	COMSOL Multyphysics	Total hip arthroplasty	Fluid pressure, film thickness, and displacement	Simplified walking condition	Static load	−	FE	Gao et al. [41]	Specific condition	−
Teoh et al. [110]	ABAQUS	Total hip arthroplasty	Von Mises stress, wear	Simplified walking condition	3D load	Brand et al. [76]	FE	Brand et al. [76]	Stance phase divided into 16 phases	−
Udofia and Jin [40]	ABAQUS v. 5.8–9	Hip resurfacing	Contact pressure, displacement, film thickness, and film pressure	Simplified walking condition	Static load	Chan et al. [111]	FE	Chan et al. [111]	Full cycle	−
Uddin [112]	ANSYS v. 12	Total hip arthroplasty	Contact pressure and von Mises stress	Simplified walking condition	Static load	Bennett and Goswami [113]	No motion	−	Peak loading	−
Uddin and Chan [114]	ANSYS v. 17.1	Total hip arthroplasty	Contact pressure, von Mises stress, and equivalent strain	Simplified walking condition	Static load	−	No motion	−	Peak loading	−
Uddin and Zhang [52]	ANSYS	Total hip arthroplasty	Contact pressure, principal stress, and wear	Simplified walking condition	3D load	Bergmann et al. [63]	FE-AA-IER	Bergmann et al. [63]	Full cycle divided into 32 phases	−
Vogel et al. [53]	ABAQUS	Total hip arthroplasty	Displacement and equivalent strain	Simplified walking condition	Static load	Bergmann et al. [115]	No motion	−	Specific condition	−
Wang and Jin [55]	Not mentioned	Total hip arthroplasty	Film thickness	Simplified walking condition	1D load	ISO 14242–1:2002 [74]	FE	ISO 14242–1:2002 [74]	Full cycle	−
Wang et al. [116]	ABAQUS v. 6.8–1	Hip resurfacing	Contact mechanics	Simplified walking condition	Static load	Heller et al. [117]	No motion	−	Peak loading	−
Wang et al. [118]	Not mentioned	Total hip arthroplasty	Film pressure and fluid pressure	Physiological walking condition	3D load	ISO 14242–1:2002 [74]	FE-AA-IER	ISO 14242–1:2002 [74]	Full cycle	−
Wu et al. [119]	Not mentioned	Total hip arthroplasty	Wear	Simplified walking condition	2D load	Saikko et al. [120]	FE	Saikko et al. [120]	Stance phase divided into 16 phases	−

The adoption of walking conditions in previous studies has been carried out by considering the loading components, both 3D load [75], 2D load [99], 1D load [41], and static load [81]. Simplification is made from the 3D load which considers loading from x-, y-, and z-axes, to only loading from x- and y-axes, or loading from y-axis. While the simplification is carried out by considering loading from the y-axis, the computational simulation results do not change significantly and can be considered a valid simplification. This is because loading from the y-axis dominates the resultant force that works when humans walk.

For the adoption of walking conditions on motion components, previous studies have considered several types, ranging from FE-AA-IER [68], FE [75], and no motion [81]. Simplification of motion components affects the contours location of computational simulation. If the adoption of motion components with FE-AA-IER or FE, then the contours of simulation results can be found to change along with the progress of walking cycle. Meanwhile, if the adoption of motion components with no motion type, the contours of the simulation results are only found in one location along with the progress of walking cycle. It was found that the adoption of motion with FE-AA-IER was all carried out simultaneously with the adoption of loading component with a 3D load so that it could represent walking conditions were close to actual conditions, as performed by Affatato et al. [57], Fialho et al. [47], and Nithyaprakash et al. [97].

Simplification of the cycle for an adopted walking condition has been carried out previously by dividing the walking cycle into 16 [69], 20 [68], 25 [99], 28 [47], 32 [59], and 41 [43] phases. Some do not adopt the full cycle component only by considering a part of the walking cycle in the stance phase as done by Maxian et al. [69], Teoh et al. [110], and Wu et al. [119]. Some studies also only adopted walking condition at specific cycle times during peak loading such as the work of Krepelka and Toth-Taşcău [70], Uddin [112], and Wang et al. [116]. However, some studies adopt the complete cycle component without simplification, an example can be found in Basri et al. [38, 54], Gao et al. [56], and Kang et al. [46].

The simplification of adopted walking condition is also influenced by the finite element model used to study couple bearings. The use of a three-dimensional finite element model allows researchers to adopt physiological walking conditions without any simplification of the loading, motions, and cycle components, as done by Affatato et al. [57], Kang et al. [46], and Mellon et al. [95]. Unfortunately, the use of the axisymmetric finite element model makes the adoption of walking condition to be simplified by not considering the motion component because of the impossibility of adoption, as was done by Ammarullah et al. [50], Jamari et al. [65], and Saputra et al [104]. However, by using a three-dimensional finite element model without axisymmetric simplification, many researchers still simplify the adoption of walking conditions by not considering the motion component that can be found in the literature by Barreto et al. [73], Cilingir [77], and Heiink et al. [45].

**Figure 5:**
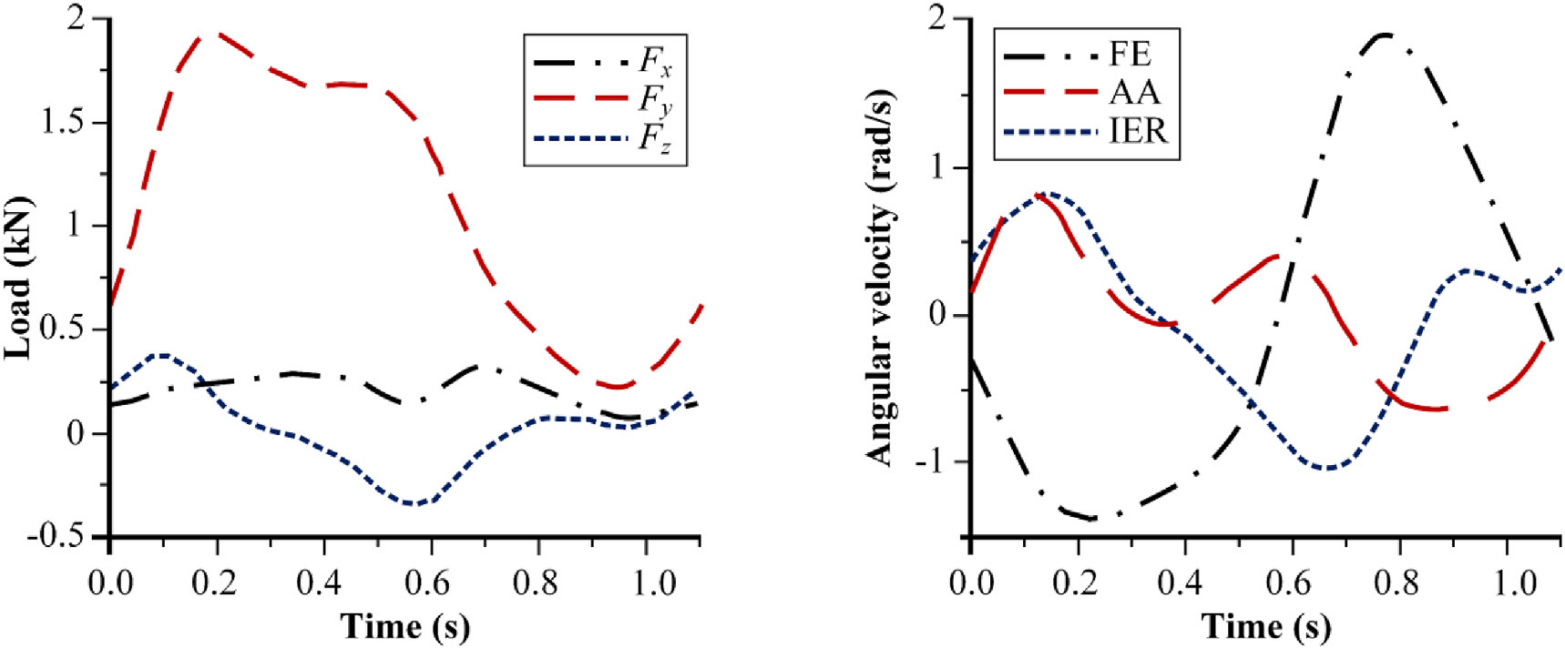
Walking condition used by Gao et al. [56].

**Figure 6:**
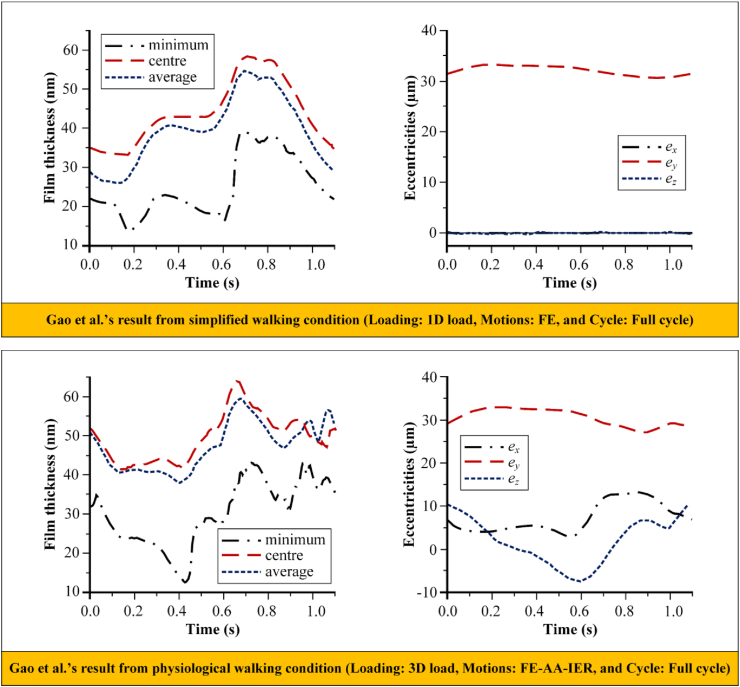
Gao et al's results from simplified and physiological walking gait cycle [56].

Simplification of adopted walking condition can certainly affect the results of computational simulation. Using walking condition data from Bergmann et al. [63] shown in Figure 5, Gao et al. [56] conducted a study comparing simplified walking condition (Loading: 1D load, Motions: FE, and Cycle: Full cycle) and physiological walking condition (Loading: 3D load, Motions: FE-AA-IER, and Cycle: Full cycle) to analyse hydrodynamic lubrication on metal-on-metal bearing of total hip arthroplasty. Figure 6 shows Gao et al.’s result for film thickness and eccentricities in two different walking gait cycles. It can be seen that there is a significant difference between results from simplified and physiological walking condition. This simplification can be fatal because it can affect data analysis and conclusions drawn by researcher. Computational simulation studies to examine bearing of hip joint implant under walking condition are strongly recommended following physiological human hip joint. If the simplification is necessary, it should be considered as minimal as possible to avoid misinterpretation of the results.

**Figure 7:**
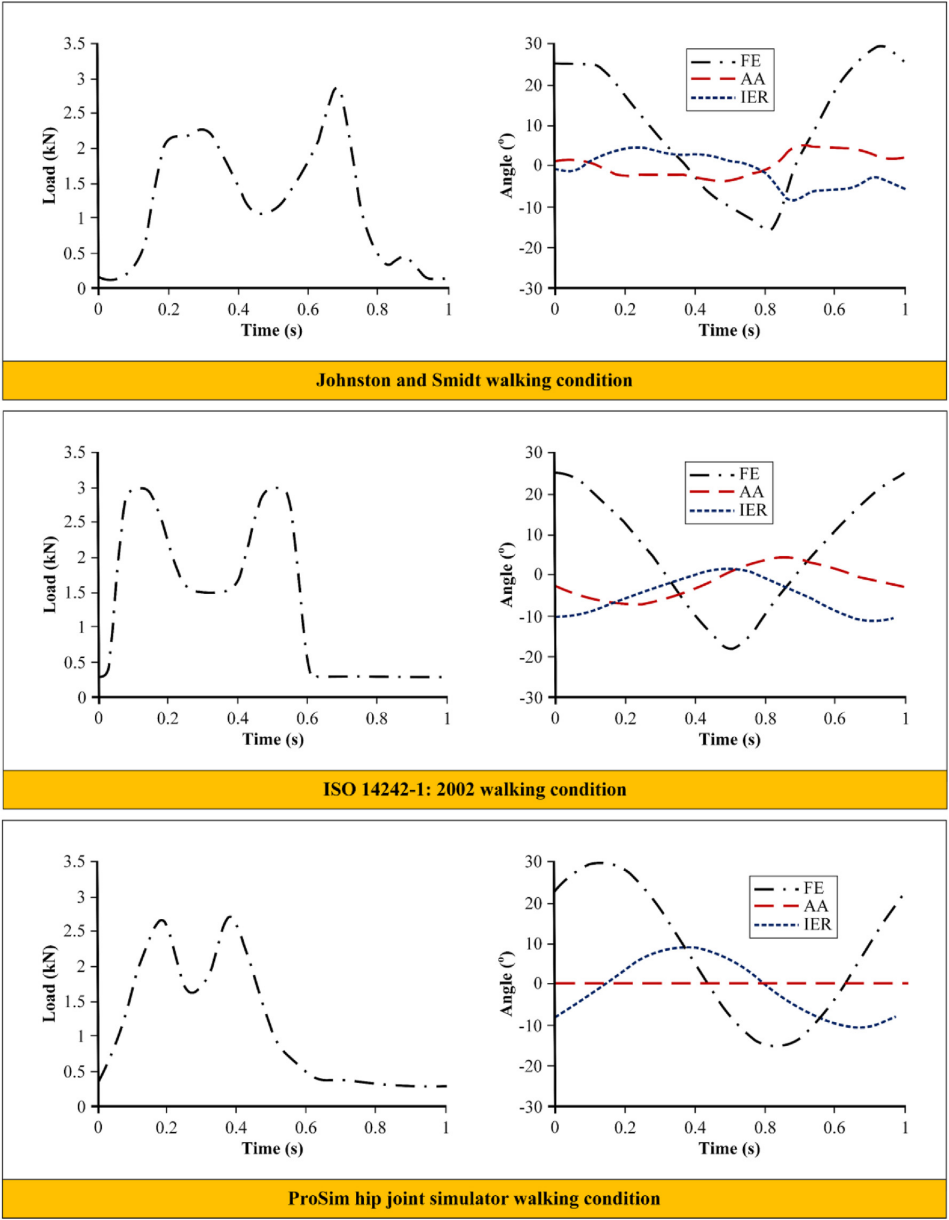
Three different walking condition used by Liu et al. [85].

**Figure 8:**
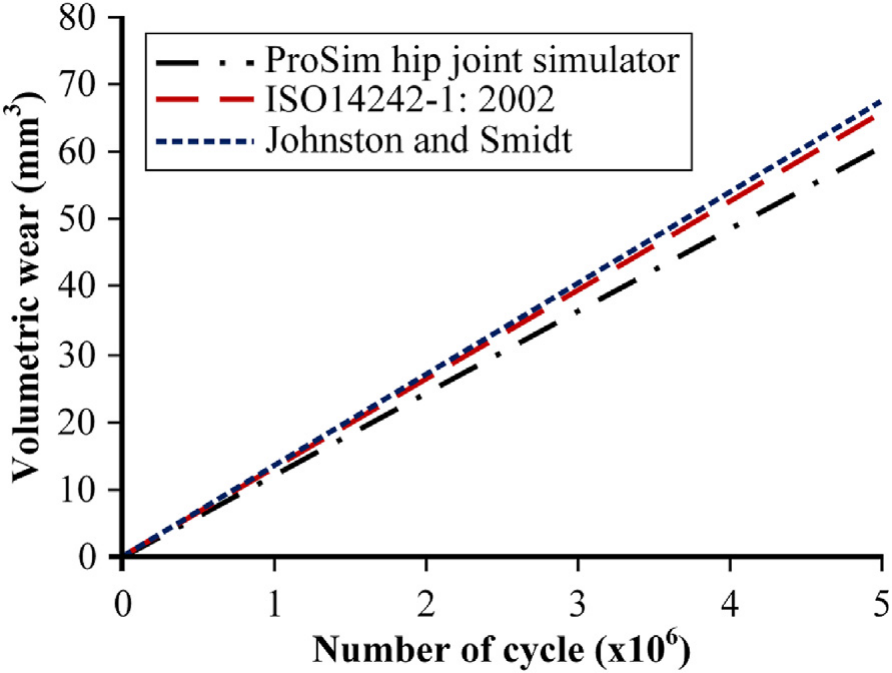
Liu et al.’s results from three different walking condition references [85].

Differences in walking condition reference can also affect the results obtained from computational simulation studies, even though they are both simplified or physiological walking conditions. Liu et al. [85] studied effect of walking condition reference to wear prediction on metal-on-polyethylene bearing of total hip arthroplasty. This research was conducted by adopting walking condition from three different references, respectively from Johnston and Smidt [80], ISO 14242-1: 2002 [74], and ProSim hip joint simulator [86] described in Figure 7. The results indicate that differences in walking condition reference can affect volumetric wear prediction, although not significant, with the highest using walking conditions from Johnston and Smidt, second from ISO 14242-1: 2002 and the lowest from ProSim hip joint simulator. Full description of Liu et al.’s results is shown in Figure 8.

## 4 Research gap and future research

Considering at research on artificial hip joint's bearing over the past 30 years with computational simulation approach, many researchers have used simplified walking condition as described in Table 3, on loading, motions, and cycle components. To provide more realistic computational simulation results according to human hip joint physiological during walking, further studies without simplification need to perform in the future. With the development of hardware technology, hardware for computational simulation will be even better that can be used to analyse various complex situations more realistically, even on low specs, thus simplification of walking condition is not needed.

Walking condition has been adopted by many researchers mostly still using “normal” condition with unspecific subjects [38, 54, 67, 102]. Subject specification is very influential from various aspects, such as abnormal [121], age [122], body mass index [123], gender [124], race/ethnic [125], religion [126], diseases experienced [127], and profession [128]. Adopting walking condition from a specific subject is crucial to the development of artificial hip joint for specific subjects. Research and production of hip joint implants, especially in bearing component, majority carried out in “normal” condition, and less considering specific subjects.

Apart from that, in daily human life, most common activities are walking. However, there are still many other activities, such as sit down/get up [63], load transfer [129], stairs up/down [117], ladder up/down [129], lift [129], carry [129], stumble [130], knee bend [129], stand on 2-1-2 legs [63], sports activities [131], religious activities [126], and other activities. It is also important to investigate the future walking condition to develop bearing on hip joint implant through a simulation approach closer to actual human daily life.

## 5 Conclusions

Many researchers have conducted several studies in developing bearing of artificial hip joints, both total hip arthroplasty and hip resurfacing through computational simulation approach using finite element method to avoid various obstacles from clinical study, experimental testing, and mathematical formula. Over the past 30 years, researchers have adopted walking condition to investigate bearing for obtaining results in solid, fluid, or both domains. Unfortunately, to alleviate heavy computational process, various studies have simplified walking conditions, whether on loading, motions, or cycles that can affect the results and lead researchers to misinterpret the results. Adoption of walking conditions of specific subjects needs to be done to develop medical implants for better results so as to minimize implant failures that require revision surgery. Considering other human activities should also be contemplated in conjunction with normal walking to obtain more realistic results.

## Declarations

### Author contribution statement

All authors listed have significantly contributed to the development and the writing of this article.

### Funding statement

The research was supported by World Class Research UNDIP number 118-23/UN7.6.1/PP/2021.

### Data availability statement

No data was used for the research described in the article.

### Declaration of interest's statement

The authors declare no conflict of interest.

### Additional information

No additional information is available for this paper.
